# Expanding Nature’s storytelling: extended reality and debiasing strategies for an eco-agency

**DOI:** 10.3389/fpsyg.2023.941373

**Published:** 2023-08-31

**Authors:** Cristina M. Reis, António Câmara

**Affiliations:** CENSE – Center for Environmental and Sustainability Research, CHANGE - Global Change and Sustainability Institute, NOVA School of Science and Technology, NOVA University Lisbon, Caparica, Portugal

**Keywords:** extended reality, pro-environmental behavior, debiasing, media psychology, storytelling, econarrative, eco-agency

## Abstract

Communication in sustainability and environmental sciences is primed to be substantially changed with extended reality technology, as the emergent Metaverse gives momentum to building an urgent pro-environmental mindset. Our work focuses on immersive econarratives, supported by virtual and augmented realities, and their potential to favor an improved relationship with the environment. Considering social aggregation dynamics and cognitive bias, this article intends to (1) make the case for a new environmental narrative; (2) position extended reality as privileged settings to sustain this narrative; and (3) suggest that this storytelling should be informed by Nature’s empirical evidence, i.e., ecosystem data. We see this as a chance to think this Metaverse with an embedded environmental consciousness, informed by behavior-change research.

## 1. Introduction

As the COVID-19 pandemic began, we navigated through an “Infodemic” (World Health Organization [WHO], 2020) of misinformation, against which some agents worked hard to prevent a potentially dramatic aggravation of the health crisis. This situation ignited public debates, informed political and private agendas, (Barua et al., 2020; Romer and Jamieson, 2020), and raised awareness over the importance of media governance and fact-checking (Seaton et al., 2020). Moreover, while some audiences were striving to understand the meaning of *exponential* or the impact of their individual role on the overall outcome, others continued to miss a crucial point: collective behavior is at the core of overcoming humanity’s environmental issues, including COVID-19.

The pandemic is part of a syndrome of human consequences (Barouki et al., 2021; Rillig et al., 2021), which also includes climate change. To have the scientific community addressing these two concerns together is recognized as critical: there is hope that doing so will reinforce the urgency of global action and behavior change toward the environment (Rillig et al., 2021). Furthermore, there is an important common ground: both climate change and the impacts of COVID-19 have been characterized as Low-Probability High-Consequence (LP-HC) events. We can learn from the pandemic experience by understanding how people perceive and act to reduce LP-HC risks (Botzen et al., 2021).

Frequently used reasons for poor judgment are related to cognitive biases and other distortions of thinking (Kahneman et al., 2021). In an attempt to increase the necessary public response toward environmental factual information, climate scientists and policymakers have long tried increasing volume and amount of information, with a low level of success (Stoknes, 2014). This growingly urgent need to broaden public discourse and engagement in addressing the environmental crisis requires new strategies, e.g., integrating ecosystem services and natural capital into mainstream economic policy; reaching out to larger audiences with dynamic scenarios and ecosystem service-based computer games (Costanza et al., 2014, 2017).

Environmental narratives have been mostly directed to the cognitive component of our attitudes, which has been proven insufficient (Stoknes, 2014). A direct discourse using real-time ecosystem data and extended reality (XR)^1^ technologies could be more effective. Our research aims at exploring those possibilities via the Internet of Things^2^ (IoT) and artificial intelligence (AI), building upon studies on public participation and environmental monitoring (Gouveia et al., 2004), climate communication strategies (Stoknes, 2014), approaches to psychological biases (Kahneman et al., 1982, 2021; Botzen et al., 2021; Korteling et al., 2021; Sulik et al., 2021), and public policies (Lechanoine and Gangi, 2020).

Extended reality is used as a tool by multidisciplinary researchers and practitioners, enhancing the public access to new opportunities, such as entertainment within the gaming industry (Cipresso et al., 2018; Slater et al., 2020), innovative health practices (Slater and Sanchez-Vives, 2016; Andrews et al., 2019; Rizzo and Bouchard, 2019), and *factual* information (Slater et al., 2020; Harris and Taylor, 2021; Markowitz and Bailenson, 2021).

Our focus on XR has been strengthened by recent conversations around the idea of an open Metaverse,^3^ which should foster environmental narratives. Further to the approach throughout traditional media, XR can shift the paradigm of environmental science communication by favoring an augmented experience based on scientific evidence. These new forms of econarrative,^4^ present in the evolving digital layers,^5^ contribute to establishing a direct relationship between audience and ecosystem data, potentially favoring the development of an eco-agency.^6^

## 2. Changing the environmental narrative

The collection of essays “Environment and narrative: new directions in econarratology” (James and Morel, 2020) is grounded on two presuppositions: stories about the environment and of how it is experienced are mutually and significantly influenced by each other; there is opportunity for scholars to revisit their understanding of the environment and rethink those stories. Additionally, the essays explore the term econarratology, defined as “the paired consideration of material environments and their representations and narrative forms of understanding,” debating how narrative theory along cognitive science can support an improved understanding of readers’ interactions with environmental storytelling, making the case for a revision of narrative models.

In one of the essays, Ursula K. Heise brings Kate Rigby to the discussion, who identifies Lisbon’s 1,755 earthquake as a trigger to “shift cultural perceptions from God’s agency to that of Nature, which enabled the modern concept of *natural disaster* and legitimated helping disaster victims.” and continues by pointing out that this model does not fit our present scenario: we have changed perspectives, as we need to take responsibility for the human actions associated with climate change, which influence the natural disasters such as the Covid-19 pandemic (Carrington, 2021).

We need urgent action on climate change, and interpretation is key to how we bring order and meaning to our lives (Bruner, 2003). Susceptible to fake news, misinformation and disinformation, cognitive biases challenge our rational thinking and faultily influence our decision making (Lechanoine and Gangi, 2020), as language can reinforce stereotypes and the projection of social biases onto others (Fiske, 1993). Climate anxiety, particularly in young people, has been associated with their perception of being condemned to not having a future, feeling a sense of abandonment by those in charge, governments and adults (Hickman et al., 2021). Information should inspire, not impair the audience. Creating opportunities for a direct observation of empirical evidence may serve a communication that is not only purposeful but in itself sustainable.

Despite the current amount of information, we still struggle with *psychological climate paradox* (Stoknes, 2014), that is, we are still missing the prioritization that these environmental concerns demand, a lack of response that is usually related to economic interests and financial crisis, psychological and cultural barriers, among others. Lifting psychological barriers could be attempted, via positive framing of climate messages or simplified choices supported by nudging (Stoknes, 2014), which could tighten the relationship between environmental policies and related public involvement. Citizen engagement is crucial to the resolution of our environmental issues, and addressing cognitive biases has been pointed as a fundamental approach to improved strategies (Stoknes, 2014; Lechanoine and Gangi, 2020; Berenbaum, 2021; Botzen et al., 2021). There are several identified biases, and some are said to possibly have evolutionary adaptative value—failing to understand the meaning of COVID-19’s *exponential* growth, and the threat it represented, is clearly maladaptive (Berenbaum, 2021).

According to aggregation dynamics, we know individual psychological processes can become group-level phenomena (Sulik et al., 2021). Furthermore, cognitive biases always create error, often shared errors, which suggests that reducing psychological biases could improve judgment (Kahneman et al., 2021). Research on biases mitigation is extensive, pointing to solutions such as creating models of psychological involvement and interaction supported by behavioral strategies (Stoknes, 2014), or using serious games for biases such as confirmation bias, fundamental attribution error and social projection.

## 3. Augmented observation, closer experience

We propose enhancing Nature’s voice by exploring combinations of empirical environmental storylines—participants could observe and interact with ecosystem regeneration data of urban bee colonies, sea forests or endangered species, visualized and auralized using XR. Other approaches could involve gaming narratives allowing for individual or social interactions—simple players’ actions could affect air’s oxygen level, water quality, food supplies, or population’s health.

A recent literature review establishes the need for more scientific evidence regarding retention and transfer of bias mitigation interventions (Korteling et al., 2021). The increasing technology support to XR research on how participants experiment, move, direct their attention, or learn (6DOF head-mounted displays, mixed reality interfaces, haptic devices, eye-tracking systems, and 5G-enabled mobile phones) provides a pathway to long-term analysis of new econarratives: continuingly presenting environmental data in dedicated user-friendly layers, with frequent interactions and corresponding outputs, allows measuring number of visualizations, level of engagement or content sharing in other media. This will involve considerations toward ethics and data privacy in XR, already under discussion (Slater et al., 2020).

Digital technology allowed for new ways of telling and responding to stories (Rose, 2021). The idea of a Metaverse, favored by 5G and new technologies, opens new paths beyond established media. Available throughout different screens (movies, TV, computers, tablets, phones), most visual storytelling media remain frame-enclosed, in front of the viewer. Moving past this framed playground enables the creation of new stories and forms of narrative: altering the content changes our relationship with it (Riggs, 2019).

Extended reality presents an opportunity for the establishment of an independent, more intimate relationship with storytelling. Research has shown that Interactive Digital Narratives (IDN)^7^ significantly increase the level of users’ engagement, when compared to a non-interactive VR environment (Irshad and Perkis, 2020). Serious gaming is also promising for behavior change (Schuller et al., 2013; Dijkman et al., 2015; Gounaridou et al., 2021), and immersive experiences are being considered effective in both eliciting empathy and changing attitudes (Bailenson, 2018; Herrera et al., 2018; Hargrove et al., 2020; Cummings et al., 2021).

An investigation focusing on building long-term empathy (Herrera et al., 2018) suggests that narrative-based and mediated perspective-taking interventions are more effective at increasing self-reported empathy than interventions without any perspective-taking tasks. The research consisted of two experiments studying empathy toward people in homeless situation, considering several behavioral measures (e.g., agreement with proposition, signing proposition). Its results reinforce the potential of immersive technologies in understanding human social behavior—beliefs, attitudes, values, and social influences processes (Ryan et al., 2019). Recent research, such as the Threshold Model of Social Influence in Virtual Environments, (Ryan et al., 2019) and the Behavioral Framework of Immersive Technologies (Wienrich et al., 2021), propose foundations on how virtual settings can support behavior change interventions. Within the Framework Behavioral Spheres (Scurati et al., 2021), our approach expands through: *the affective dimension*, by bridging and nurturing a direct, closer connection between audience and Nature; *the rational dimension*, by providing an opportunity for learning through empirical evidence; *the practical dimension*, through predefined strategies, such as participant monitoring, data collection, or other forms of engagement.

Immersive experiences also allow raising awareness, through empathy and cooperation, on a wide range of critical issues that can only be solved by broad consensus, with different audiences and innovative strategies. Some people are motivated by doing what they think is right for the environment, whereas others are interested in buying a collection of rare Nature NFT’s,^8^ or earning awards for their eco-friendly attitudes. XR will still present limitations with populations facing economic or political struggles, such as poverty or censorship, but enabling a significantly improved response to environmental issues may help reducing inequalities (Botzen et al., 2021).

In addition, XR permits a direct observation of scientific evidence, e.g., ecosystems data can provide different outputs and illustrate human intervention, the benefits of conservation or the damaging consequences of their aggravated state. The theory of psychological proximity infers that the ability to empathize is directly related to the proximity of our own experience to that of others (Hargrove et al., 2020). XR can offer access to information with higher transparency and various POV. Though we are still in the presence of a mediated relationship—there is a medium and an output—it aims to establish a closer connection to nurture the participants’ engagement and the value they attribute to empirical evidence.

In general, XR studies show promise in environmental education (Ahn et al., 2016; Markowitz et al., 2018; Filter et al., 2020; Chirico et al., 2021; Fauville et al., 2021), Nature connectedness and pro-environmental behavior (Soliman et al., 2017; Klein and Hilbig, 2018; Breves and Schramm, 2021), by allowing the user to viscerally experiment distinct points of view (POV) (Herrera and Bailenson, 2021). Research is advancing over environmental education and ecological behavior, relating exploration and learning of complex concepts (Markowitz et al., 2018), bias, and ethics (Slater and Sanchez-Vives, 2016; Slater et al., 2020).

## 4. Extended Nature

With the support of ecosystem data and AI, we propose to picture Nature as a personified communicative agent, via different roles leading to distinct XR experiments: there is a realm of possible econarrative structures, where Nature can assume contrasting or complementing roles, moving far beyond a simple narrator.

– *Co-creative:* Scientific data can inform the virtual representations of ecosystems impacted by human intervention. Drafted along with its non-human characters (Donly, 2017), material environments and their representations (James and Morel, 2020), this storytelling is inspired by existing relationships within multiple ecosystems.
– *Participant:* Focusing on the desired sense of cooperativeness among individuals, we address collective impact by testing its possibilities in influencing the user’s experience, when applied to Immersive Interactive Narratives (IIN). We consider exploring the possibilities of including Nature as a participant.
– *Agent:* The relevance of dialog to cooperativeness also comes to mind, if we consider the example of several narrators in one story as a way to avoid the sovereignty of a protagonist over the others (Donly, 2017). Supported by its data and AI in its interaction, Nature may be represented by an agent (as opposite to a human Avatar) while remaining present as an active narrator, equal protagonist in the story.

Human empathic ability toward non-human species is variable: according to the anthropomorphic stimuli hypothesis (Miralles et al., 2019), human ability to connect emotionally with other organisms is mostly dependent on the number of external features that can intuitively be perceived as homologous with theirs. XR has proven its empathic abilities, and our proposal aims to build on these to better understand humans’ empathic relation with Nature, informing the debate around anthropomorphic interpretations, and potentially unveiling new ways of promoting a sense of closeness to living beings perceived as less similar to humans.

We are particularly interested in an interdisciplinary approach to promote a pro-environmental mindset by:

- Tackling behavioral barriers with exploratory experiences that defy the audience sense of time, space and self (Wienrich et al., 2021).
- Testing interactive immersive narratives, particularly focused on positive emotions and/or constructive actions.
- Exploring individual agency and collective impact. For instance, in a study on promoting attitude and behavioral change in plastic consumption in VE, a visual representation of the consumption has showed better results when compared to a mere display in numerical terms (Chirico et al., 2021).

## 5. Nature as a co-creator (case study)

Studies indicate a high association between urbanicity and mental health issues, and the benefits of Nature connectedness are often discussed, for instance toward stress recovery (Ulrich et al., 1991) and capability of nurturing a pro-environmental behavior (Nisbet et al., 2009; Otto and Pensini, 2017; Barrera-Hernández et al., 2020). These are also considered within immersive scenarios (Calogiuri et al., 2018; Klein and Hilbig, 2018; Breves and Heber, 2020). Connecting citizens with local Nature is of the essence to raise awareness about current environmental issues.

Picture a capital city with a park of 100 hectares, in which lies a botanical garden and forest. By installing an array of networked low-power wireless temperature sensors, we enable visitants to visualize, through their mobile phones, the park’s temperature distribution in real time. Displaying this virtual layer on top of the botanical species shows how temperature varies drastically from one location to another, along with the corresponding trees, vegetation, and other species. The user interface allows time-travel into the future, based on current climate change predictions, and present the negative impact of increasing temperatures on botanical diversity. Gamification can be added to make the exploration more interesting: collective effort to care for the ecosystem or scavenger hunt with easter eggs (which can drive younger audiences to visit and care for their urban parks). Missions can support the development of eco-agency, including favorite spot nominations to observe the audience relationship with the surroundings.

## 6. Discussion

If the Metaverse represents a new way of navigating the world, XR must be explored as a new path for a relationship with Nature. Our perspective highlights the opportunity of establishing a place for Nature’s discourse, with storytelling constructs and narrative pathways laying the grounds for further research approaches. We propose new econarrative constructs, supported by ecosystem data, considering them an essential part of an iterative process toward a sustainable society. This approach has the potential to establish a relationship with environmental information with less mediation than traditional settings. XR is perceived as a tool to strengthen alternative forms of interactive econarratives, explore debiasing strategies, and assess their impact on behavior change to continuingly improve this iteration. By facilitating a direct observation of Nature’s narrative, XR can contribute to an improved understanding of the environment, which can lead to the development of an eco-agency. Our research suggests that expanding Nature’s storytelling will significantly change environmental science communication, which ultimate goals are to inform and educate for a sustainable behavior.

## Data availability statement

The original contributions presented in this study are included in the article/supplementary material, further inquiries can be directed to the corresponding author.

## Author contributions

CR: conceptualisation and writing. AC: supervision. Both authors approved the submitted version.

## Footnotes

1 Collective term for technologically mediated experiences (Bertrand et al., 2018) by virtual and augmented reality technologies (VR/AR), where VR is a completely virtual immersive setting, AR if in the presence of computer-generated perceptual information in the physical world, and Mixed Reality (MR) as a superimposition of both VR/AR, where physical and digital elements interact.

2 A network of physical objects with sensors, processing capacity and multiple technologies, that can connect, exchange data with other systems and devices.

3 Adopted from the novel Snow Crash (Stephenson, 1993), it’s considered an immersive world created by VR/AR layers built on top of the physical world, allowing for new ways of interaction between people and new forms of accessing information, through the use of technical equipment (such as headsets). Being very recent and susceptible to a certain degree of appropriation (ex. Facebook became Meta), this concept should remain open to new nomenclatures.

4 Also eco-narrative(s), perceived as an approach to storytelling that endeavors to compose with its non-human characters (Donly, 2017).

5 Driving from the concept of mirror world (Gelernter, 1991), i.e., a digital representation of the real world, these are understood as separate elements digitally expressed.

6 Considered as the understanding of having the capacity to produce an effect, by decision, intervention, or action, in the environment.

7 Narratives used within digital media that respond to user input. As we are focusing our research on extended reality technologies, we also refer to these as Interactive Immersive Narratives (IIN).

8 Non-fungible token is digital data (ex. an image) stored in a blockchain technology who secures its ownership, therefore allowing for its commercialization.
